# Heart failure and cognitive impairment: A narrative review of neuroimaging mechanism from the perspective of brain MRI

**DOI:** 10.3389/fnins.2023.1148400

**Published:** 2023-03-27

**Authors:** Tong Li, Xiangyuan Bao, Lin Li, Rui Qin, Cuicui Li, Ximing Wang

**Affiliations:** ^1^Department of Radiology, Shandong Provincial Hospital Affiliated to Shandong First Medical University, Jinan, China; ^2^School of Radiology, Shandong First Medical University, Taian, China

**Keywords:** heart failure, cognitive impairment, brain lesions, brain magnetic resonance, MRI

## Abstract

Both heart failure (HF) and cognitive impairment (CI) have a significant negative impact on the health of the elderly individuals. Magnetic resonance imaging (MRI) can non-invasively detect functional and structural variations in the heart and brain, making it easier to explore the connection between the heart and brain. According to neuroimaging studies, HF patients have a higher chance of developing CI because they have a variety of different types of brain injuries. To examine how HF and CI are influenced by one another, English-language literature was searched in the Web of Science, PubMed EMBASE (OVID), PsycInfo, and Scopus databases. The search terms included “high-frequency,” “brain function,” “brain injury,” “cognition,” “cognitive impairment,” and “magnetic resonance imaging.” Normal brain function is typically impaired by HF in the form of decreased cerebral perfusion pressure, inflammation, oxidative stress, and damage to the BBB, resulting in CI and subsequent HF. Early pathophysiological alterations in patients’ brains have been widely detected using a range of novel MRI techniques, opening up new avenues for investigating the connection between HF and CI. This review aims to describe the pathogenesis of HF with CI and the early diagnostic role of MRI in the heart-brain domain.

## 1. Introduction

A common tendency in population growth around the world is aging. Due to their significant incidence in the senior population, heart failure (HF) and cognitive impairment (CI) are receiving increasing amounts of attention. HF is a common clinical disease characterized by a decrease in the blood supply of the heart due to anatomical or functional problems with the heart. In China, studies on the epidemiology of HF have revealed an increase in the prevalence rate among people over 35, from 0.9% in 2000 to 1.3% in 2019 (Hao et al., 2019). CI refers to one or more impairments of cognitive function that affect daily or social abilities for various reasons, such as memory and learning. It covers all stages, from mild CI to dementia. Studies have confirmed that up to 50% of HF patients develop a certain level of CI, and 10% of them have more serious symptoms (Vogels et al., 2008; Almeida et al., 2012).

Ischemic cardiomyopathy and hypertension are considered major causes of HF and are associated with cognitive function (Schmidt et al., 1991). Decreased systolic function of the heart and blood redistribution are symptoms of HF, which is frequently a subsequent condition to myocardial infarction (Roy et al., 2017; Jinawong et al., 2021). The fundamental etiology of brain functional injury is thought to be the decrease in cerebral perfusion pressure brought on by decreased cardiac output and inflammation worsened by oxidative stress (Harkness et al., 2014; Cannon et al., 2017). Although anatomical abnormalities in the brain were linked to biomarkers of myocardial injury and cardiac failure, only older people with poorer cognitive reserves displayed cognitive deficits (Feola et al., 2013; Hjelm et al., 2014). HF and CI patients with poor self-care abilities witness a significantly decreased quality of life and higher rates of readmission and mortality (Yang et al., 2022). Participants in previous studies have not always been categorized according to the subtype of HF. Consequently, the phrase “heart failure” broadly refers to a variety of cardiac failures.

In recent years, an increasing number of studies have verified that CI frequently occurs in patients with HF. Magnetic resonance imaging (MRI) technology and its derivatives can detect spontaneous brain activity and investigate image biomarkers, which offer the foundation for early identification of CI. However, the specific pathogenesis of HF has not yet been clarified, and the correlation between HF and cognitive function needs to be further researched by MRI. Understanding the impact of the heart on the brain in HF patients as well as the function of MRI in early diagnosis in the heart-brain area are the main objectives of this review. Two separate researchers examined the English literature in the PubMed EMBASE, Web of Science, PsycInfo, and Scopus databases after receiving systematic review training. The search terms included “high-frequency,” “brain function,” “brain injury,” “cognition,” “cognitive impairment,” and “magnetic resonance imaging.”

## 2. Pathogenesis of HF with CI

### 2.1. Cerebral perfusion pressure decreased

To keep the brain functioning normally, there must be enough oxygen and glucose in the blood—approximately 20 and 25%, respectively, of the body’s energy (Sabayan et al., 2016). Endothelial dysfunction in HF patients may result in aberrant cerebrovascular reactivity (the brain’s blood vessel response to high amounts of carbon dioxide), decreased automatic brain regulation, and other symptoms (Zuccalà et al., 1997). Cardiovascular illnesses are significantly influenced by hypoperfusion, which is caused by poor cardiac output and low blood pressure. Aging and vascular risk factors both enhance the likelihood of developing Alzheimer’s disease and chronic cerebral hypoperfusion (Georgiadis et al., 2000). Patients with HF often have decreased myocardial contractility and cardiac output, which lead to decreased cerebral perfusion pressure. Compared with age-matched healthy controls, HF patients’ resting cerebral blood flow (CBF) was 31% lower (Vogels et al., 2008). Patients with mild and moderate HF experienced lower middle cerebral artery blood flow (47.3 and 56.1 cm/s, respectively) (Scherbakov and Doehner, 2018). Other complications, such as sleep apnea, diabetes, hypertension, and depression, are exacerbated by a decrease in CBF (Lorenzi-Filho et al., 2002). There is evidence of reduced CBF in the bilateral hippocampus, parahippocampal gyrus, and right posterior cingulate gyrus cortex in people with HF (Gruhn et al., 2001; Sabayan et al., 2015), which is typically associated with AD. Reduced CBF makes HF patients more susceptible to brain parenchymal injury, particularly gray matter injury (Muller et al., 2011). Various cortical areas linked to executive cognitive performance are affected by reduced gray matter volume (Amanzio et al., 2021). In conclusion, brain tissue loss and dysfunction caused by brain hypoperfusion can speed up CI in people with HF. Further correlation studies are required since the impact of local CBF reduction on molecular cognitive performance has not yet been fully understood.

### 2.2. Inflammatory response and oxidative stress

Interdependence between the inflammatory response and oxidative stress is common and is linked to brain dysfunction following heart disease (Zhu et al., 2007). Myocardial damage in HF patients usually results in increased inflammation and the immune response (Gill et al., 2010), and low perfusion caused by decreased heart function also results in cerebral inflammation (Akiguchi et al., 1997). In the cortex and hippocampus of HF mouse models, the expression of inflammatory genes such as Toll-like receptor 4 (TLR4), tumor necrosis factor-α (TNF-α), and interleukin-6 (IL-6) increases dramatically (Hong et al., 2013). TNF-α is a key regulator of the brain’s proinflammatory response, increasing neurotoxicity through glutamate secretion by neurons and leading to cell damage and death (Perry et al., 2001), affecting synaptic plasticity as well as brain learning and memory function. A brain with decreased cognitive function results from IL-6’s increased expression of beta-secretase and aberrant increases in beta-amyloid formation and deposition. These inflammatory cytokines pass through the blood–brain barrier (BBB) and enter the brain, where they trigger an inflammatory reaction that impairs cognition.

### 2.3. Damage to the blood-brain barrier

The BBB is crucial in preventing metabolic waste and harmful substances from passing into the central nervous system through the blood circulation (Liori et al., 2022). The pathophysiology of CI linked to HF is greatly influenced by aberrant BBB function, which includes pericellular breakdown, endothelial cell activation, and an excessively tight linkage between endothelium and pericellular (Ritz et al., 2013). The primary cell type that make up the blood–brain barrier, endothelial cells, has been demonstrated to be vulnerable to harm from elevated intracellular calcium ion levels (Doyle et al., 2008). BBB dysfunction worsens microvascular damage worse, fosters secondary inflammation, damages vascular tone, leads to stenosis of the cavity, and causes tissue ischemia (Vancheri et al., 2020), which promotes contact between neurotoxic proteins and neurons.

### 2.4. The neurohumoral axis

The neurohumoral axis of patients with HF plays a role in cognitive and structural changes in the brain. The sympathetic nervous system and the renin-angiotensin-aldosterone system quickly come into action when the output decreases to restore perfusion pressure and stop dehydration and traumatic bleeding (Gruhn et al., 2001). The hormone cortisol, which is linked to stress, has an impact on cognitive function. Temporary high cortisol levels have been linked to impairments in cognitive function. Other studies have shown that persistently elevated cortisol levels may result in atrophy in specific brain areas because of reduced neurogenesis (Chetty et al., 2014). The brain’s hypothalamus, hippocampus, and amygdala showed the highest levels of glucocorticoid receptor expression, whereas the hippocampus, amygdala, and prefrontal cortex margin showed the highest amounts of halocorticoid receptor expression (Gallina et al., 2014). Cortisol levels are much higher in HF patients with depression and CI than in HF patients without these symptoms (Huffman et al., 2013), which raises the possibility that cortisol levels in HF may impact how rapidly CI develops.

### 2.5. Alimentary deficiency

An increasing number of studies have found that there is a close relationship between nutritional deficiency and CI. Researchers have found that nutritional deficiencies can lead to decreased attention, memory, and cognitive function in humans with HF (Stewart et al., 2015). Patients are prone to nutritional deficiency due to increased body consumption and changes in systemic metabolism, and nutritional deficiency will also aggravate the degree of HF (Alosco et al., 2013).

### 2.6. Depressive disorder

According to research, depression is linked to greater levels of CI, structural alterations in the brain (Sheline et al., 1999), inflammation (Schiepers et al., 2005), and neurohormone biomarkers, which are also present in HF patients. CI performance also improved in patients with depression who received medication (Halvorsen et al., 2012). Further research is necessary to fully understand the function of depression in HF and CI.

## 3. Advances in brain imaging of HF and CI

### 3.1. Conventional magnetic resonance imaging

The approach to craniocerebral evaluation most frequently utilized currently for the clinical diagnosis and care of HF patients is conventional MRI scanning. MRI can detect whether there are ischemic lesions, infarction lesions, brain atrophy, and other structural changes in patients’ brains. The two main contributing factors to HF are thought to be ischemic heart disease and hypertension (Vogels et al., 2007a). It is believed that overall brain shrinkage and aberrant cerebral vascularization are the root causes of cognitive decline in HF patients (Schmidt et al., 1991). White matter hyperintensity (WMH) was connected to sorrow and anxiety, and atrophy of the medial temporal lobe was connected to cognitive problems such as memory loss and executive dysfunction in HF patients (Vogels et al., 2007a). Reduced hippocampal volume is present in HF patients, which may contribute to depression and short-term memory loss (Woo et al., 2015). According to the studies above, patients with HF and CI will have brain structure changes. Conventional MRI is only applicable to lesions with significant brain structure and cannot fully identify the pathological mechanism. With this limitation, it should be combined with other more effective MRI-derived techniques for observing changes in brain microstructure and function.

### 3.2. Functional magenetic resonance imaging

Functional magnetic resonance imaging (fMRI) uses computer refinement of multiple images to measure changes in neural signals caused by changes in brain structure and neural activity. fMRI combines the advantages of the high-resolution anatomic imaging capability of conventional MRI with the specificity of blood flow dynamics to directly and accurately observe the large activity processes of the brain during the implementation of cognitive tasks. Lower cardiac function results in decreased task-related brain area efficiency and reduced verbal working memory task performance in older patients with cardiovascular illness (Irani et al., 2009). HF patients showed a reduced neural activation response to Valsalva in a number of autonomic and motor control regions, including the cerebellar cortex and vermis, amygdala, hypothalamus, ACC, left insula, left putamen, and bilateral posterior central gyrus (Ogren et al., 2012; Song et al., 2018). These regions may influence emotional transmission, attention, perception, and long-term memory through structural and functional connections in brain tissues such as the visual cortex. Brain MRI imaging analysis in HF patients has shown that brain dysfunction is connected to cardiac remodeling and results in less gray matter in the primary motor cortex and hippocampus. It might be affected by daily activities and lessen depression symptoms in HF patients (Suzuki et al., 2013). These results support the notion that HF patients experience CI and anatomical alterations to the brain. fMRI has a diverse set of applications in brain cognitive research and neural activities, but much in-depth research is still needed to elucidate the changes in higher brain functions.

### 3.3. Structural magnetic resonance imaging

In recent years, a variety of image analysis and detection techniques have been applied in structural magnetic resonance imaging (sMRI) research, reducing the influence of subjective manipulation on the experiment, including voxel-based morphometry (VBM) (Frey et al., 2021). VBM is a technique that involves reflecting subtle changes in gray and white matter volume or density at the unit level to measure brain structure images accurately. Surface-based morphometry (SBM) can extract a variety of anatomical parameters, such as cortical surface area, cortical thickness, and the gyri index, which measures the morphological characteristics of the cerebral cortex more comprehensively (Naegel et al., 2022).

Structural magnetic resonance imaging showed brain atrophy and other static tissue abnormalities in patients with CI. Patients with HF had a greater frequency of medial temporal lobe atrophy than healthy controls (Frey et al., 2021). Many studies have used sMRI to explore the relationship between CI and brain structural changes. The entorhinal cortex and hippocampus of the medial temporal lobe were the first areas of the brain to exhibit cerebral atrophy (Du et al., 2004), and it progressed in line with the Braak stage. Gray matter loss affects the middle temporal gyrus, fusiform gyrus, parahippocampal gyrus, and temporal pole in people with chronic HF. Moderate medial temporal lobe atrophy is where the loss starts, and it progresses to the rest of the temporal lobe (Li et al., 2011). The degree of medial temporal lobe atrophy was assessed using the visual Scheltens score with great sensitivity and specificity (Kilimann et al., 2014). Selective attention impairment, verbal and visual memory deficits, and other cognitive skills are all tightly associated with medial temporal lobe atrophy in HF patients (Frey et al., 2018). CI in HF patients is associated with loss of gray matter density in the lateral, anterior cingulate, and medial frontal cortex. They have been discovered to be connected to psychological functions such as emotion, pain, and cognitive control (Merkler et al., 2019). In fact, both HF and coronary heart disease exhibit the same pattern of brain injury (Mueller et al., 2020). However, investigations have shown that there is gray matter loss in specific areas in patients with HF and that this loss is more widespread than that in patients with coronary heart disease and in unaffected controls. Reduced gray matter density in sizable brain regions such as the hippocampus, prefrontal cortex, and precuneus may enable CI (Vogels et al., 2007b). In addition, the decrease in GMD was correlated with the decrease in left ventricular ejection fraction and the increase in NT-proBNP.

When referring to T2-weighted images or T2 fluid decay inversion recovery sequences, the term “WHM” refers to high signal expression in deep cerebral or periventricular white matter. Small artery disease-induced coronary microvascular dysfunction is a common pathogenic mechanism of WMH and HF (Camici et al., 2020). As a specific variation in CI concomitant with HF, the speed of information processing and executive function have been linked to an increase in WMH (Tan et al., 2022). The severity and duration of HF are associated with decreased cardiac function, which can have a significant impact on the deep white matter of the brain (Vogels et al., 2007a). According to Vogels et al. (2007b) research, individuals with HF who did not also have stroke, dementia, or depression were more likely to have WMH on brain MRI. WMH refers to high signal expression in deep cerebral or periventricular white matter on T2-weighted images or T2 fluid decay inversion recovery sequences. WMH was still substantially more common in HF patients even if IHD, age, and other affecting factors were excluded (Vogels et al., 2007a). These results imply that individuals with HF display localized brain abnormalities on sMRI that are comparable to those in patients with CI, with cognitive function being impacted by gray matter density reduction and white matter microstructure loss. However, most of the studies on brain atrophy have focused on the medial temporal lobe, not other brain regions.

### 3.4. Cerebral perfusion imaging

Magnetic resonance perfusion imaging is an examination technique that detects microcirculation distribution and hemodynamic changes in brain tissue to assess local tissue and function. It can be used to assess CBF and metabolic status. HF can lead to cerebral hypoperfusion and decreased metabolic activity, resulting in decreased cognitive function. Arterial spin-label perfusion imaging and magnetic resonance perfusion imaging were the imaging methods employed to assess CBF (Chandra et al., 2019). Arterial spin-labeled perfusion imaging is a non-invasive method for measuring arterial blood flow based on nuclear magnetic flow markers. Changes in local cerebral perfusion can be assessed without the use of radiation or contrast agents. In patients with HF, reduced CBF and brain tissue injury were found in many areas, such as the frontal vascular bed, parietal lobe, occipital cortex, hippocampus, thalamus, and cerebellar region (Woo et al., 2003, 2009). In addition, the decrease in CBF was significantly lateralized, with the main decreased areas in the right cortex and diencephalon. According to studies, perfusion imaging can diagnose Alzheimer’s disease and moderate CI with 87 and 67% accuracy, respectively (Lacalle-Aurioles et al., 2014). Cerebral perfusion imaging has strong repeatability and can effectively reflect changes in cerebral hemodynamics. However, there are few studies on the use of MRI perfusion imaging in people with HF and CI, and its clinical utility is not frequently used.

## 4. Conclusion and future directions

There is growing evidence that HF and CI are linked and brought on by HF-induced brain damage. Patients with HF will have apparent CI as well as changes in brain structure, function, and metabolic status due to decreased cerebral perfusion pressure, an inflammatory response, oxidative stress, and BBB breakdown. New MRI techniques such as sMRI, fMRI, and brain perfusion imaging are conducive to further research on the early pathophysiological changes of CI in HF patients. Brain sites with tissue damage in HF patients include the cingulate gyrus, hypothalamus, hippocampus, insula, brainstem, amygdala, and cerebellar regions. Attention deficit hyperactivity disorder, learning disabilities, memory loss, language impairments, and decreased visuospatial performance are all closely associated with HF. It is important to note that neuroimaging studies on HF with CI are still in the beginning stages. Most studies are retrospective, and the application of new techniques needs to be expanded. To better understand the connection between HF and CI, extensive cohort studies on HF, cognitive function, and MRI will be required in the future. Additionally, early magnetic resonance diagnostics play an important role in achieving early detection, diagnosis, and treatment.

## Author contributions

TL: writing—original draft. XB: writing—review and editing. LL: conceptualization. RQ: data curation. CL and XW: supervision and writing—review and editing. All authors contributed to the article and approved the submitted version.
